# Differences in IVD characteristics between low back pain patients and controls associated with HIZ as revealed with quantitative MRI

**DOI:** 10.1371/journal.pone.0220952

**Published:** 2019-08-22

**Authors:** Christian Waldenberg, Hanna Hebelka, Helena Brisby, Kerstin Magdalena Lagerstrand

**Affiliations:** 1 Department of Medical Physics and Techniques, Sahlgrenska University Hospital, Gothenburg, Sweden; 2 Institute of Clinical Sciences, Sahlgrenska Academy, University of Gothenburg, Gothenburg, Sweden; 3 Department of Radiology, Sahlgrenska University Hospital, Gothenburg, Sweden; 4 Department of Orthopaedics, Sahlgrenska University Hospital, Gothenburg, Sweden; University College London, UNITED KINGDOM

## Abstract

**Background:**

Magnetic resonance imaging (MRI) can provide objective continuous intervertebral disc (IVD) measures in low back pain (LBP) patients. However, there are limited studies comparing quantitative IVD measures of symptomatic and asymptomatic individuals.

**Purpose:**

This study aimed to investigate possible differences in IVD tissue composition in patients with chronic LBP and controls using quantitative MRI and correlate IVD measures with the phenotype High-Intensity Zone (HIZ).

**Methods:**

The lumbar spine of 25 LBP-patients (25-69y, mean 38y, 11 males) and 12 controls (25-59y, mean 38y, 7 males) was examined with T2-mapping on a 1.5T MRI scanner. The mean T2-map value and standard deviation were determined in three midsagittal IVD slices and five sub-regions dividing each IVD in the sagittal plane. The distribution of T2-map values over the IVD was also determined with histogram analysis (Δμ = distribution width).

**Results:**

When compared to controls, patient IVDs displayed lower values for all metrics, with significant differences for the T2-map value, standard deviation (p = 0.026) and Δμ (p = 0.048). Significantly different T2-map values were found between cohorts in the region representing nucleus pulposus and the border zone between nucleus pulposus and posterior annulus fibrosus (p = 0.047–0.050). Excluding all IVDs with HIZs resulted in no significant difference between the cohorts for any of the analyzed metrics (p = 0.053–0.995). Additionally, the T2-map values were lower in patients with HIZ in comparison without HIZ.

**Conclusions:**

Differences in IVD characteristics, measured with quantitative MRI, between LBP patients and controls were found. The T2-differences may reflect altered IVD function associated with HIZ. Future studies are recommended to explore IVD functionality in relation to HIZ and LBP.

## Introduction

Low back pain (LBP) causes more global disability than any other condition [1] and is a well-documented source of chronic disability for both genders in the working age [2]. Intervertebral disc (IVD) degeneration involves biochemical changes (*i*.*e*. loss of proteoglycan and water content), structural changes (*i*.*e*. annular tears, Schmorl's nodes), and metabolic changes (*i*.*e*. reduced matrix synthesis) [3] and is strongly associated with LBP [4]. However, IVD degeneration is also common among asymptomatic individuals [5] and this limits the clinical relevance of IVD degeneration used for targeting the source of LBP.

Research has aimed to establish the relationship between IVD degeneration and LBP through gross morphological IVD measures. Also, Modic changes [6] as well as the presence of High-Intensity Zones (HIZs) [7] have been pointed out as specific degenerative markers in the spine and may, as such, be associated with new specific IVD metrics. First defined by Aprill and Bogduk in 1992 [7], HIZ was described as a small well-defined area with a high-intensity signal on T2-weighted magnetic resonance images in the posterior annulus of the IVD which today is thought to represent inflamed IVD disruption possibly extending deep into the IVD [8].

In the last decade, various quantitative MRI methods have been developed that provide new objective and continuous IVD measures [9–11]. However, no specific pain marker for LBP has yet been found [12], probably due to the inclusion of overlapping phenotypes in the study cohorts and the use of gross quantitative IVD measures in the analysis. Detailed characterization of the IVD in terms of quantitative measures of hydration, tissue composition and tissue heterogeneity in combination with a systematic evaluation of asymptomatic and symptomatic individuals as well as sub-grouping into promising phenotypes may reveal underlying patterns of LBP. The IVD hydration, tissue composition and tissue heterogeneity can be determined using detailed T2-mapping analysis, e.g. texture and histogram analysis. For example, Waldenberg et al. recently demonstrated the feasibility of histogram analysis in LBP-patients and showed a large spread in heterogeneity metrics for IVDs with similar degeneration grade [9]. Hence, comparison of such detailed quantitative metrics between symptomatic and asymptomatic individuals could potentially be of value in the diagnosis of LBP.

The aim of this work was to study possible differences in IVD tissue composition between patients with chronic LBP and controls using detailed T2-mapping analysis and correlate possible differences in IVD measures with the phenotype HIZ.

## Material and methods

### Cohort/Intervertebral discs

Twenty-five LBP patients (11 males, 25–69 years, mean 38 years, median 36 years) and 12 controls (7 males, 25–59 years, mean 38 years, median 31 years) were included. All patients suffered from chronic LBP (minimum six months of continuous pain) of non-specific character, i.e. patients with apparent LBP causes, other than IVDs with herniation, bulging or degenerated IVD disease, were not included in this study. Two IVDs were excluded due to severe degeneration causing segmentation difficulties. The control cohort included in this study were originally recruited for another study that investigates possible effects of axial loading on the lumbar spine. In this study, the T2-maps from the unloaded examinations were further analyzed.

All procedures performed in studies involving human participants were in accordance with the ethical standards of the institutional and/or national research committee and with the 1964 Helsinki declaration and its later amendments or comparable ethical standards. The study was approved by Regional Ethics Review Board Gothenburg. Oral and written informed consent was obtained from all individual participants included in the study.

### Magnetic resonance imaging protocol

The lumbar spine (L1 to S1) of all individuals were examined *in vivo* on a 1.5 T MRI scanner (Siemens Magnetom Aera, Erlangen, Germany). All subjects were examined with conventional sagittal T1-weighted (T1W) and T2-weighted (T2W) imaging (Table 1). Additionally, all subjects were scanned with quantitative T2-mapping technique which is sensitive to the IVD water and collagen content [13]. This technique constructs a ‘map’ in which the tissue-specific quantitative T2-relaxation time is determined pixel-by-pixel and displayed as varying pixel intensity. The T2 mapping involved scanning with a multi-echo spin-echo based sequence. Calculation of the T2-value was performed with the Siemens MapIt analysis tool, assuming that the resulting T2 decay of the acquired echo-train exhibit a pure mono-exponential decay of the form S(t) = S0exp(−t/T2). The acquired T2-maps were evaluated in phantom measurements to have an error of <7% for T2-values representative for IVDs of Pfirrmann grade 3 (data not shown).

**Table 1:** MRI parameters for T1W, T2W and T2-map scanning of LBP patients and controls.

	T1W (TSE)	T2W (TSE)	T2W (TSE)	T2-map (MESE)
Imaging plane	Sagital	Sagital	Axial	Sagital
Repetition time (ms)	480	3500	4862	1400
Echo time (ms)	9	95	97	multi (11.1, 22.2, 33.3, 44.4, 55.5, 66.6, 77.7, 88.8)
Echo train length	3	14	17	8
Slice thickness (mm)	3.5	3.5	3.5	3.5
Slice gap (mm)	0.7	0.7	0.4	0.7
Number of averages	2	1	2	1
Pixel bandwidth (Hz)	235	180	195	220
Flip angle (degree)	150	150	150	180
Acquisition matrix	320×224	384×288	320×256	256×256
Reconstruction matrix	320×320	384×384	320×320	256×256
Field of view (mm^2^)	300×300	300×300	200×200	220×220

T1W = T1 weighted; T2W = T2 weighted; TSE = Turbo Spin Echo; MESE = Multi-Echo Spin-Echo

### Image analysis

The degeneration of each IVD was evaluated on T2W images according to the Pfirrmann scale [14] by a senior radiologist with 15 years of experience. The degeneration was determined on two occasions, blinded to previous evaluation, separated in time by one month. In order to determine inter-observer agreement, a subsample of the IVDs was graded by a second radiologist with four years of experience. Since HIZs potentially could affect regional T2-map values [15] any existing HIZ were visually identified by an experienced radiologist on either sagittal or axial T2 weighted images, according to April and Bogduk [7].

### Image post-processing

**Fig 1:**
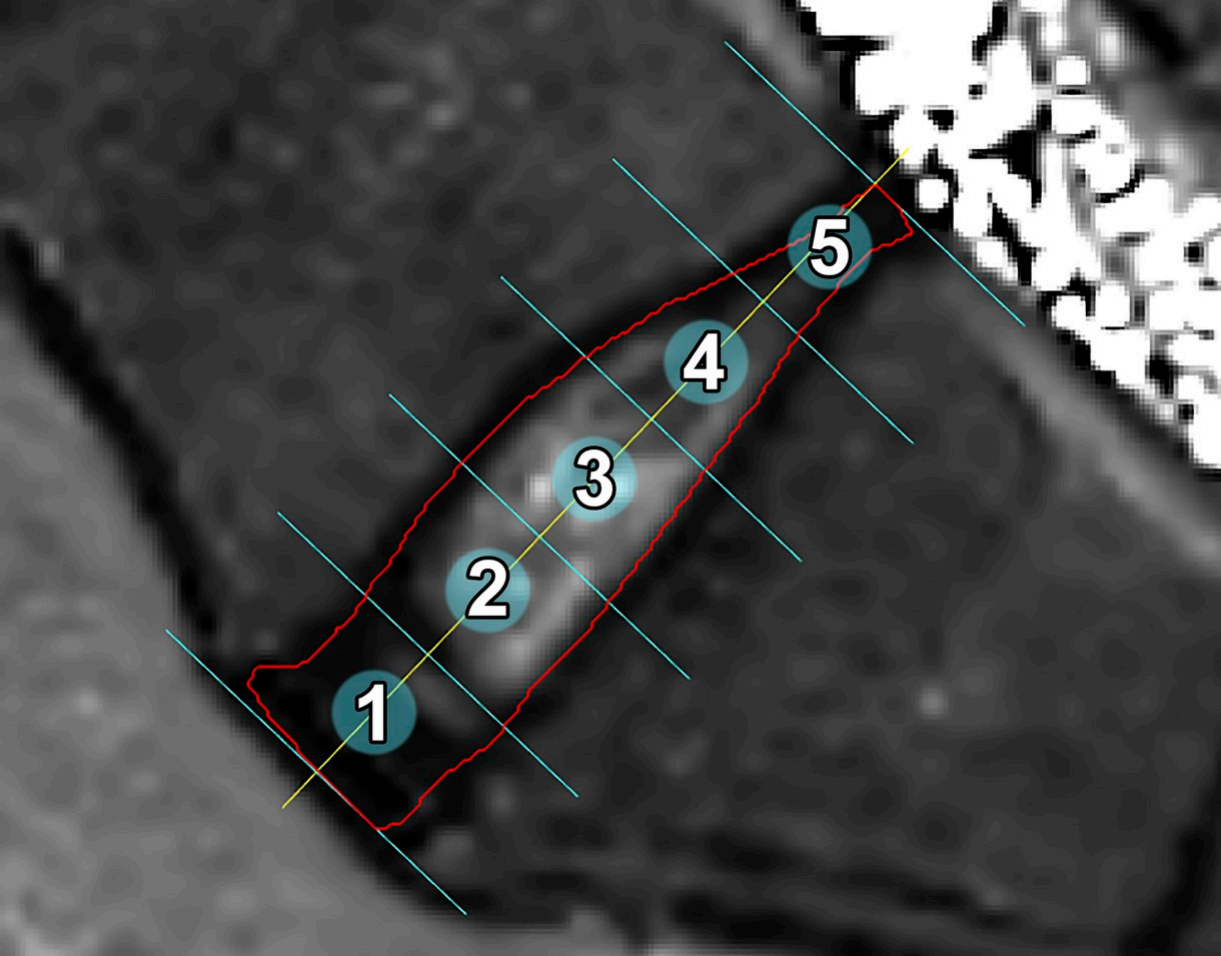
Example of segmentation of an IVD. Example of the segmentation of a T2-map performed on L5-S1 IVD. Each segmentation was divided into five equally sized sub-regions ranging from 1 (anterior) to 5 (posterior) using an in-house developed software.

Post-processing of the image data was performed using the MATLAB software R2016b (Mathworks, Natick, Massachusetts, U.S.A.). Using semi-automated segmentation based on a combination of a region-growing algorithm and manual adjustment, each IVD included in this study was outlined on three consecutive mid-sagittal morphological T1W-images (Fig 1). During segmentation, the outer contours of the IVDs were manually adjusted. Care was taken to exclude tissue not associated with the IVD. Instead, if there was doubt, the contour was contracted to minimize the risk of including foreign tissue inside the “regions of interest” (ROI). The segmented ROIs were then transferred onto the T2-map. As the size and position of the T1W image field of view did not match the T2-map, the ROIs were rescaled and positioned to match the corresponding T2-map. This was done automatically via the patient position and pixel spacing information stored in the DICOM image tags. The measurements were performed by an image analysist who had three years of experience from MR-image analysis and previously received extensive training supervised by a senior radiologist specialized on spine diagnostics with 15 years of experience. The Intra-rater variability was determined by repeating the measurement on a subset of the IVDs. The inter-rater variability was determined with a second reader on a different set of images. The second reader was a radiologist with 4 years of experience from MR-image analysis who received extensive training supervised by a senior radiologist. All readings were approved by the senior radiologist.

To evaluate the impact of degeneration regionally on the IVD, each ROI was divided into five, in the sagittal direction, equally sized sub-regions stretching from posterior to anterior IVD (Fig 1). This is an analysis method similar to the one conducted by Stelzeneder et al. and Nilsson et al. [10, 16, 17]. The observer was blind to the individuals’ identity during segmentation and later analysis.

Detailed analysis of the IVD-tissue was performed by calculating metrics that had the potential to characterize differences in IVD degeneration, *i*.*e*. mean, SD and histogram distribution of the T2-map values over the segmented ROIs and sub-regions. To enable robust and consistent evaluation of histogram distribution of the T2-map values as well as reduce the risk of potential miscalculation of the IVD metrics, all T2-maps were truncated to remove bright noise before being further analyzed.

The mean T2-map value was determined by calculating the average of all T2-map values of the voxels inside each ROI or sub-region in all three slices. The SD was determined by calculating the standard deviation of all T2-map values of the voxels inside each ROI or sub-region in all three slices. The distribution of the mean T2-map value and SD in the sub-regions was plotted to show the regional behavior in the cohorts. Moreover, the distribution of IVD T2-map values was evaluated using the histogram analysis method proposed by Waldenberg et al. [9]. The method uses a two-component Gaussian probability distribution model to separate the full range of IVD T2-map values into two sections. Each section reflects either the IVD annulus fibrosus (AF) or the IVD nucleus pulposus (NP). By calculating the peak separation (Δμ) of the two Gaussian components, the distinction between the two IVD tissues can be estimated and thereby the degeneration process can be followed.

The mean IVD T2-map values, SD and Δμ were plotted as a function of the Pfirrmann grade and compared between patients and controls.

An additional regional analysis of the patient measurements was performed to further investigate the influence of HIZ on IVD composition and the IVD T2-map values.

### Statistical analysis

Mann-Whitney’s U-test was performed to test statistical significance (P<0.05) between patient and control IVDs for each Pfirrmann grade and each sub-region. Significance was not tested for Pfirrmann grade 5 due to too few IVDs with this degeneration grade.

The intra- and inter-observer reliability of the Pfirrmann grading was determined with Cohen's kappa coefficient (κ). The coefficients were interpreted according to Landis and Koch [18]. Kappa values between 0.0 and 0.2 represent slight agreement, values between 0.21 and 0.40 represent fair agreement, values between 0.41 and 0.60 represent moderate agreement, 0.61 to 0.80 represent substantial agreement and values exceeding 0.81 represent almost perfect agreement.

Reliability of the segmentation measurements for intra- and inter-rater agreement was performed using intraclass correlation coefficients (ICC) with 95% confidence intervals. ICC model 2 was used with single measurement to determine the absolute agreement. The coefficients were interpreted according to Cicchetti [19]. ICC values less than 0.40 represent poor agreement, values between 0.40 to 0.59 represent fair agreement, 0.60 to 0.74 represent good agreement and values exceeding 0.74 indicate excellent agreement.

## Results

The analyzed IVDs displayed a normal distribution of Pfirrmann grades with the largest number of IVDs rated Pfirrmann grade 2 and lowest number rated Pfirrmann grade 5 (Table 2).

**Table 2:** Distribution of IVDs between Pfirrmann grades in patient and control cohorts.

Pfirrmann grade							
		1	2	3	4	5	1–5
Patients	No HIZ	12 (9.7%)	55 (44.4%)	25 (20.2%)	11 (8.9%)	1 (0.8%)	104 (83.9%)
	HIZ	0	0	8 (6.5%)	11 (8.9%)	1 (0.8%)	20 (16.1%)
	All	12 (9.7%)	55 (44.4%)	33 (26.6%)	22 (17.7%)	2 (1.6%)	124 (100%)
Controls	No HIZ	7 (11.9%)	36 (61.0%)	6 (10.2%)	5 (8.5%)	1 (1.7%)	55 (93.2%)
	HIZ	0	0	0	4 (6.8%)	0	4 (6.8%)
	All	7 (11.9%)	36 (61.0%)	6 (10.2%)	9 (15.3%)	1 (1.7%)	59 (100%)

No HIZ = IVDs with no HIZ; HIZ = IVDs with HIZ; All = sum of No HIZ and HIZ.

For the Pfirrmann grading, the intra-observer agreement was substantial (κ = 0.74) and the inter-observer agreement was moderate (κ = 0.56). For the segmentation of the IVDs, the inter- and intra-observer agreement was excellent (ICC 0.94–0.98).

**Fig 2:**
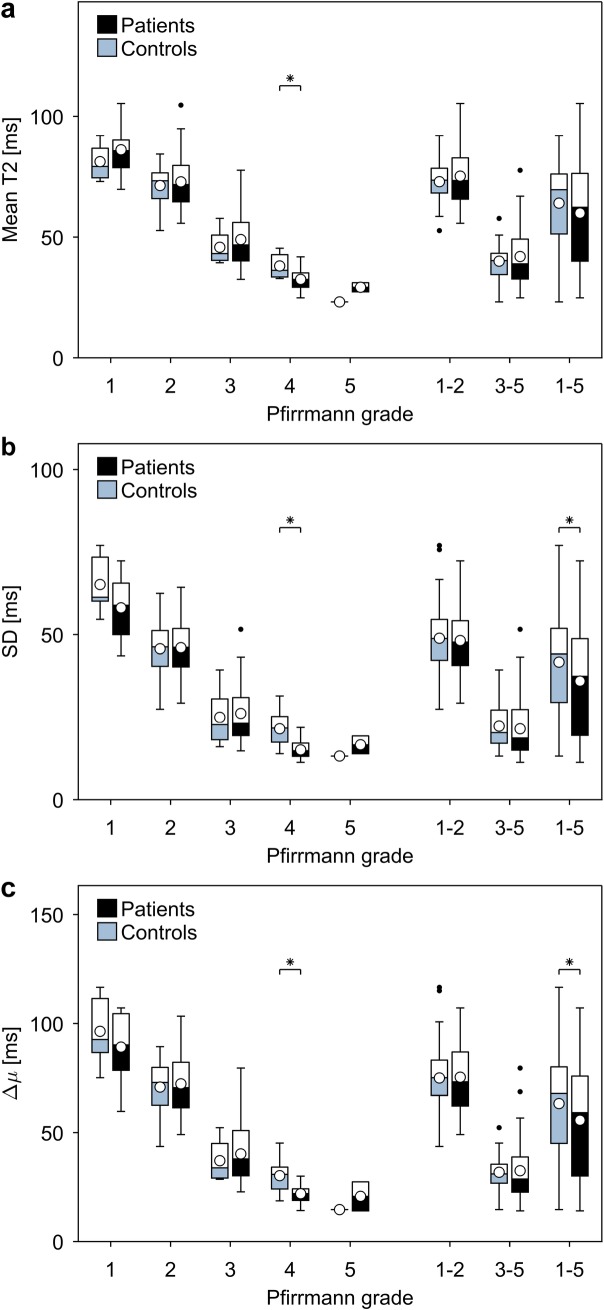
Metric values as a function of Pfirrmann grade for patient and control cohorts. Pfirrmann grade versus the **a)** mean T2-map value **b)** standard deviation and **c)** peak separation (Δμ) determined for voxel values within the IVD. The whiskers extend to the most extreme data points not considered outliers. The outliers are indicated with a black marker and the mean for each box is indicated with a white marker. Significance, indicated with an asterisk, was tested between patients (black) and controls (blue) for each Pfirrmann grade.

On a group level, patients displayed lower IVD-values for all metrics when compared to controls, with significant differences for SD (p = 0.026) and Δμ (p = 0.048), but no significant difference for the mean T2-map value (p = 0.115) (Fig 2). When IVDs with the same Pfirrmann grade were compared between the cohorts, significant differences for all metrics were found regarding Pfirrmann grade 4 (p = 0.003–0.007) (Table 3).

**Table 3:** P-values for metrics compared between patient and control cohorts.

		Pfirrmann grade							
		1	2	3	4	5	1–2	3–5	1–5
Mean T2	HIZ	0.536	0.842	0.573	**0.007**	-	0.540	0.995	0.115
	No HIZ	0.536	0.842	0.115	0.145	-	0.540	0.222	0.520
SD	HIZ	0.167	0.964	0.712	**0.003**	-	0.736	0.484	**0.026**
	No HIZ	0.167	0.964	0.355	0.090	-	0.736	0.436	0.193
Δμ	HIZ	0.432	0.913	0.654	**0.006**	-	0.995	0.655	**0.049**
	No HIZ	0.432	0.913	0.202	0.115	-	0.995	0.323	0.298
		Sub-region							
		1	2	3	4	5			
Mean T2	HIZ	0.712	0.499	0.050	**0.047**	0.312			
	No HIZ	0.689	0.869	0.313	0.300	0.053			
SD	HIZ	0.668	0.168	**0.024**	**0.035**	0.503			
	No HIZ	0.528	0.573	0.182	0.181	0.160			

HIZ = analysis include HIZ IVDs; No HIZ = analysis does not include HIZ IVDs; P-values in bold indicate significance (p<0.05).

For the entire IVD, the mean T2-map value, SD and Δμ decreased with increasing degeneration in both cohorts (Fig 2).

**Fig 3:**
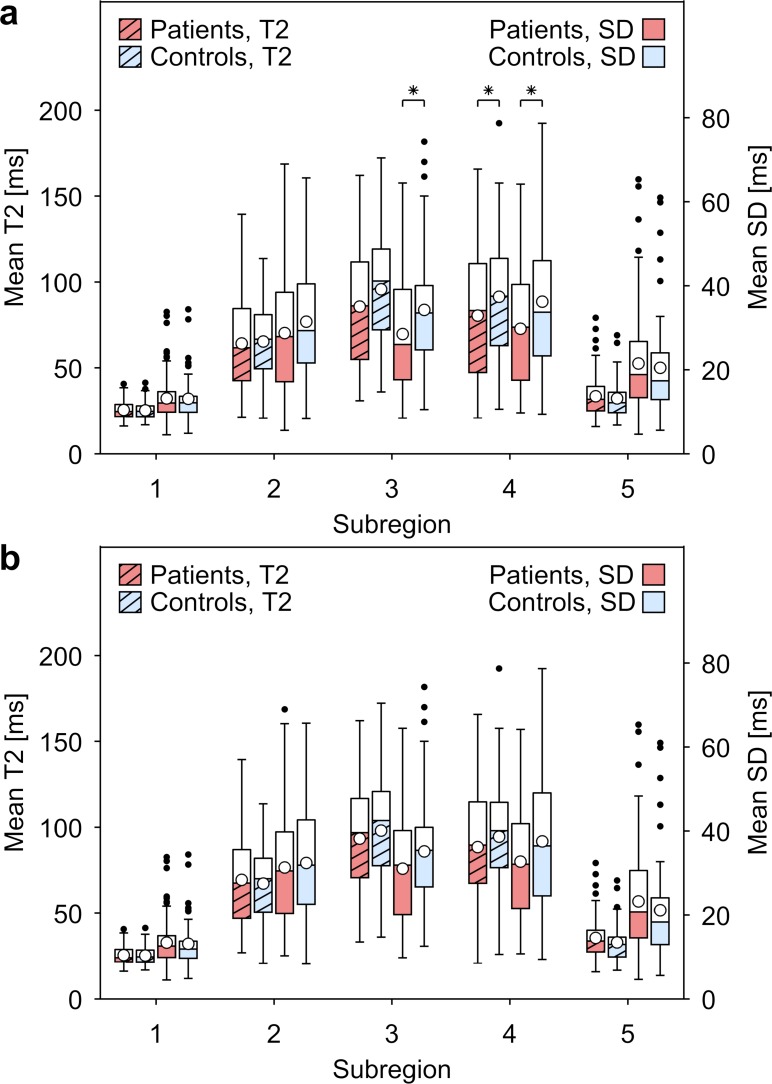
Mean T2-map values and mean SD as a function of IVD sub-region for patient and control cohorts. Sub-regions versus mean T2-map value (dashed boxes, left axis) and standard deviation (solid boxes, right axis) determined for voxel values within the IVD. An analysis was performed for **a)** all IVDs and **b)** IVDs without HIZ. The whiskers extend to the most extreme data points not considered outliers. The outliers are indicated with a black marker and the mean for each box is indicated with a white marker. Significance, indicated with an asterisk, was tested between patients (red) and controls (blue) for each sub-region.

**Fig 4:**
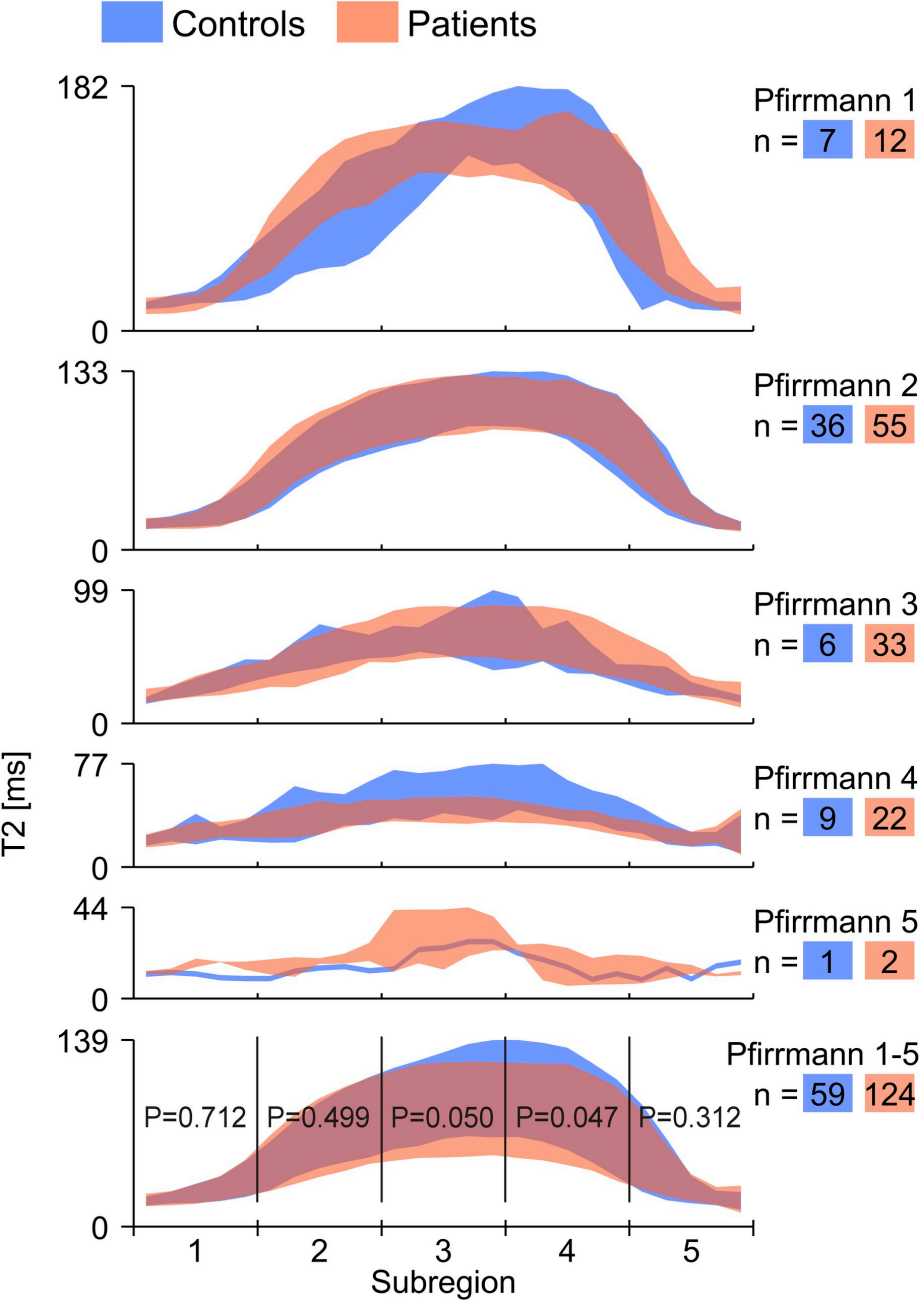
Distribution of T2-map values for IVD sub-regions in patient and control cohorts. The distribution of T2-map values for different sub-regions of the IVD ranging from 1 (anterior) to 5 (posterior) is represented as two overlapping patches of different colors. Blue represents the controls and red represent the patients. The height of each patch visualizes the mean T2-map value ± one SD. The T2-map value is indicated on the y-axis and the x-axis indicates the anterior-posterior location. The individuals are sorted into groups of different Pfirrmann grades and the number of IVDs in each group is denoted with *n*. In the bottom graph (Pfirrmann 1–5), significance was tested between patients and controls for each sub-region.

Analysis of each separate sub-region revealed significantly different SD (p = 0.024) and near significant mean T2-map value (p = 0.050) between the two cohorts for sub-region 3 (Figs 3 and 4). For sub-region 4, a significant difference was found for both SD (p = 0.035) and the mean T2-map value (p = 0.047).

A total of 20 HIZs were found in the patient cohort (16.1% of IVDs, prevalence 64.0%) and 4 in the control cohort (6.8% of IVDs, prevalence 33.3%) (Table 2). All HIZs were located in the outermost posterior parts of the IVD (sub-region 5). After excluding all IVDs with HIZ from the analysis, no significant differences between the two cohorts could longer be found for any of the analyzed metrics, neither when comparing analysis results of the entire IVD (p = 0.054–0.995) nor of individual sub-regions (p = 0.053–0.869).

**Fig 5:**
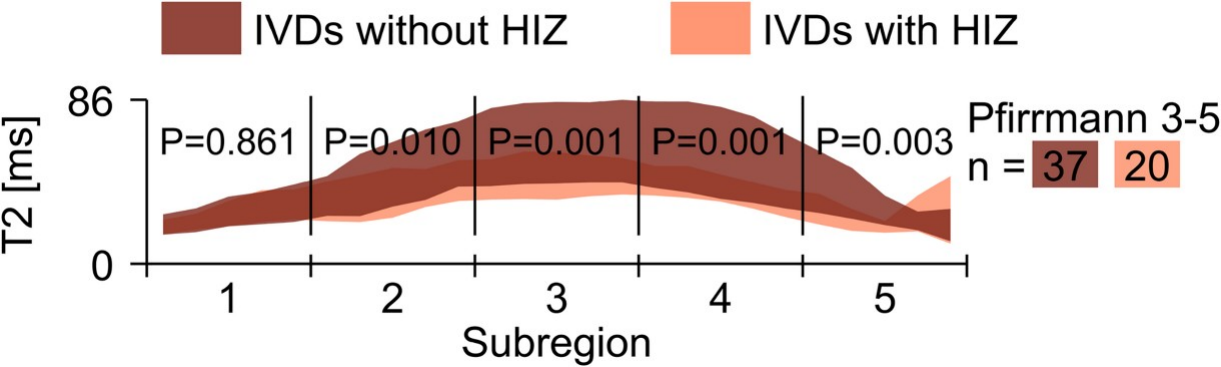
Distribution of T2-map values for different sub-regions of patient IVDs with HIZ and patient IVDs without HIZ. The distribution of T2-map values among patients for different sub-regions of the IVD ranging from 1 (anterior) to 5 (posterior) is represented as two overlapping patches of different colors. Bright red represents patient IVDs with HIZ and dark red represents patient IVDs with no HIZ. The height of each patch visualizes the mean T2-map value ± SD. The T2-map value is indicated on the y-axis and the x-axis indicates the anterior-posterior location. The number of IVDs in each group is denoted with *n*. For each sub-region, significance was tested between patients IVD with and without HIZ.

As only four IVDs in the control group had HIZ, a comparison between patient HIZ IVDs and control HIZ IVDs could not be performed. Instead, additional analysis regarding patient IVDs with and without HIZ was performed. Since all HIZs were located within Pfirrmann grade 3–5, patient IVDs with HIZ were only compared to patient IVDs without HIZ graded Pfirrmann grade 3–5. In this additional analysis, significantly lower mean T2-map values were found in four sub-regions of IVDs with HIZ when compared to IVDs with no HIZ (sub-region 1–5: p = 0.861, 0.010, 0.001, 0.001, 0.003) (Fig 5).

## Discussion

This MRI study, comparing detailed IVD characteristics between patients and controls, present small but relevant quantitative differences. When analyzing the entire IVD on a group level, significantly lower quantitative values (mean SD and Δμ) were found in the patient cohort in comparison to the control cohort, reflecting increased tissue homogeneity (SD) and reduced tissue distinction (Δμ). The presence of HIZ was associated with altered IVD characteristics and matrix composition, foremost in the NP region. When all HIZ IVDs were excluded from the analysis, no significant differences between the cohorts were found. Also, the sub-analysis of patient IVDs suggest that HIZ IVDs are different in their matrix composition, foremost regarding NP region, compared to IVDs without HIZ.

Several studies promote HIZ as a promising marker for discogenic pain [8, 20] as HIZ lesions contain antigenic NP material trapped between torn lamellae structures in AF that induce inflammation and ingrowth of vascularized granulation tissue deep into the IVD [8, 21]. However, a high prevalence of HIZ IVDs among asymptomatic individuals [22, 23] (33.3% in this study), and a wide range of reported sensitivity for HIZs and concordant pain during discography [7, 24, 25], questions the reliability and limits the use of HIZs in the diagnosis of LBP. Thus, it is plausible that the source for painful IVDs somehow is connected to HIZ, but other changes within the disc may be equally or more important.

In this study, the presence of HIZ was associated with altered IVD characteristics, mainly in the NP region. The observed T2-decrease in regions occupied by NP, probably reflecting reduced hydration in IVDs with HIZ, is in concordance with earlier studies [8, 26]. In a histologic study, Peng et al. [8] described that HIZ IVDs had markedly elevated fibrosis and matrix cell density in NP. Theoretically, this reduces the proteoglycan concentration that is responsible for the attraction of water and, thus, the level of hydration in NP [13]. Furthermore, as HIZ lesions contain clusters of nuclear cells originating from the NP, it seems valid that part of NP can prolapse and leak into AF, yielding lower T2-map values in NP as well as increased IVD homogeneity (SD) and decreased tissue distinction (Δμ). This likely explains the differences found between the two cohorts, with a higher incidence of HIZ in the patient group (Fig 4, Table 2). The additional analysis, comparing patient IVDs with and without HIZ, also supported the hypothesis, yielding higher T2-map values in NP for IVDs without HIZ (Fig 5). Moreover, IVDs with HIZ were shown to have lower T2-map values in most regions of the IVD, with the largest difference slightly off-center towards the posterior side of the IVD (Fig 5). This is in accordance with the findings of Trattnig et al. [15] who reported lower NP T2-map values in IVDs with annular tears compared to IVDs without. Similarly to our results, they also reported significant different T2-map values in the posterior annular region. In contrary, Ogon et al. [27] reported no significant difference in mean T2-map values between LBP patients and cohorts within NP, only within the posterior AF. Differences between these studies could be due to methodology issues, as they used smaller ROIs in their analysis representing NP/AF and did not report whether any HIZ existed. Using conventional MRI techniques, prior studies have also reported similar findings [28] which reinforces our findings.

All analyzed metrics correlated well with IVD degeneration, measured in terms of Pfirrmann grade, and followed the same trend. That is, they decreased with increased degeneration, suggesting that all present metrics can be used to study IVD matrix development in a detailed, objective and continuous way, providing more information regarding matrix structure and heterogeneity compared to traditional metrics. Especially the heterogeneity measures SD and Δµ, which revealed differences between cohorts even when the mean T2-map value did not, seemed to be sensitive metrics for longitudinal studies of the IVD.

The deviant metric values in the central IVD parts, shown in the current study, could reflect impaired microstructure of the IVD associated with HIZ. In order to evaluate the findings of this feasibility study and the metrics potential clinical value as pain markers, a large cohort-study with equal distributions of HIZ in matched groups of asymptomatic and symptomatic individuals is encouraged.

Reliable quantitative T2-mapping has been a long-standing challenge as the measurements can be influenced by several factors, including relaxation effects during excitation [29–31], radiofrequency flip angle variation along the slice profile [32], magnetic field inhomogeneities, and stimulated echo formation from a multi echo trains [31]. To offer a significant decrease in scan time, multi-echo sequence schemes are generally used for T2-mapping. However, such schemes are often contaminated by stimulated echoes, leading to a non-exponential T2 decay and an overestimation of the actual T2-value. The size of the contamination depends on several factors, such as pulse sequence timing, radiofrequency flip angle variation along the slice profiles and more. Therefore, we encourage quality assessments to verify the reliability of the actual T2-mapping protocol and highlight that comparisons between different sites and studies using T2-mapping techniques should be performed with caution.

### Limitations

The study included a smaller control cohort when compared to the patient cohort (12 controls versus 25 patients). This is due to the fact that the control cohort was originally recruited to a different study. However, the smaller control cohort size was accounted for during the statistical analysis. The control cohort was slightly younger (median age 31 years versus 36 years) which might partly explain the differences between the groups. However, the cohort mean age was matched and it is not likely that the clear differences in the analyzed metrics can be fully explained by the small difference in median age or other factors such as sex or BMI. It is possible that delineation of the ROI boundaries in the posterior regions of the IVDs was influenced by the detection of a HIZ. Especially the presence of vertical annular tears may aid in identifying the true edge of the posterior IVD. However, vertical tears were uncommon among the recruited individuals and in case of doubt, the contour was contracted to minimize the risk of including foreign tissue inside the ROI.

## Conclusions

We found differences in IVD characteristics, measured with detailed T2-mapping, between LBP patients and controls. The differences found between the cohorts may reflect an altered function of the IVD associated with HIZ. Future studies are recommended to further explore the IVD functionality in relation to HIZ and LBP.

## Supporting information

S1 Dataset Dataset of patient and control cohorts. (MAT)
